# Supplementary material for: Correspondence between the Compositional and Aromatic Diversity of Leaf and Fruit Essential Oils and the Pomological Diversity of 43 Sweet Oranges (Citrus x aurantium var sinensis L.)
Source: Plants (Basel). 2023 Feb 21;12(5):990. doi: 10.3390/plants12050990 (PMC10005092; doi:10.3390/plants12050990)
Supplement: Supplementary file 1 [file plants-12-00990-s001.zip › Supplemental S1_list of accessions.pdf]

Supplemental S1 Table: Names of cultivars used in this study as well as their coded identifiers and phenotypic groups.

Cultivar	Id	Group
Bisri	SRA 390	blond
Cadenera	SRA 232	blond
Cam Mat	SRA 811	blond
Fukuhara	SRA 562	blond
Hamlin	SRA 41	blond
Huan Pi Chen	SRA 567	blond
Lue Gim Gong	SRA 397	blond
Madame Vinous	SRA 551	blond
Maltaise Blonde	SRA 560	blond
Natal	SRA 398	blond
Parson Brown	SRA 43	blond
Pera	SRA 399	blond
Pinneaple	SRA 42	blond
Portugaise	SRA 400	blond
Quintela	SRA 659	blond
Ruby	SRA 402	blond
Salustiana	SRA 486	blond
Salustiana SG	SRA 403	blond
Shamouti	SRA 299	blond
Sweet	SRA 50	blond
Valencia Late SG	SRA 246	blond
Boukhobza	SRA 569	blood
Maltaise demi blood	SRA 703	blood
Maltaise demi blood SG	SRA 237	blood
Moro SG	SRA 301	blood
Petit Pierre demi blood	SRA 570	blood
Blood	SRA 545	blood
Bloodlli	SRA 243	blood
Bloodllo	SRA 406	blood
Bloodllo Moscata Cuscuna	SRA 405	blood

Sokotoro	SRA 407	blood
Tarocco	SRA 573	blood
Tarocco Chelif	SRA 539	blood
Tarocco Rosso	SRA 574	blood
Cara Cara Navel	SRA 666	navel
Fisher Navel	SRA 669	navel
Navelate	SRA 307	navel
Navelina	SRA 672	navel
Navelina SG	SRA 305	navel
Washington Navel	SRA 39	navel
Washington Navel SG	SRA 217	navel
Iaffaoui Douce	SRA 660	acidless
Sakkaria Lokum	SRA 678	acidless
